# Identifying the key characteristics of a culturally safe mental health service for Aboriginal and Torres Strait Islander peoples: A qualitative systematic review protocol

**DOI:** 10.1371/journal.pone.0280213

**Published:** 2023-01-12

**Authors:** Helen Milroy, Shraddha Kashyap, Jemma R. Collova, Monique Platell, Graham Gee, Jeneva L. Ohan

**Affiliations:** 1 UWA Medical School, University of Western Australia, Perth, Western Australia, Australia; 2 Bilya Marlee School of Indigenous Studies, University of Western Australia, Perth, Western Australia, Australia; 3 School of Allied Health, University of Western Australia, Perth, Western Australia, Australia; 4 Murdoch Children’s Research Institute, The Royal Children’s Hospital, Parkville, Victoria, Australia; 5 Mebourne School of Psychological Sciences, University of Melbourne, Melbourne, Victoria, Australia; 6 School of Psychological Science, University of Western Australia, Perth, Western Australia, Australia; Manaaki Whenua - Landcare Research, NEW ZEALAND

## Abstract

**Background:**

Mental health inequities between Indigenous and non-Indigenous populations are well documented. There is growing recognition of the role that culturally safety plays in achieving equitable outcomes. However, a clear understanding of the key characteristics of culturally safe mental health care is currently lacking. This protocol outlines a qualitative systematic review that aims to identify the key characteristics of culturally safe mental health care for Aboriginal and Torres Strait Islander peoples, at the individual, service, and systems level. This knowledge will improve the cultural safety of mental health care provided to Indigenous peoples, with a focus on Aboriginal and Torres Strait Islander peoples in Australia.

**Methods and expected outputs:**

Through a review of academic, grey, and cultural literature, we will identify the key characteristics of culturally safe mental health care for Aboriginal and Torres Strait Islander peoples in Australia. We will consider the characteristics of culturally safe care at the individual practitioner, service, and systems levels.

**Prospero registration number:**

CRD42021258724.

## Introduction

Prior to colonisation, Indigenous peoples across the globe lived healthy lives, and their mental health and wellbeing flourished for thousands of years. However, today, many Indigenous peoples face disproportionate rates of mental health challenges as the result of intergenerational trauma and the continuing experience of colonising practices and attitudes [1–4]. The contributing role of mental health services and systems to mental health outcomes is under increasing scrutiny by mental health professionals, service providers, and service users [5–7]. In turn, there is growing consensus regarding the need for culturally safe mental health care [6, 8], particularly within the Australian policy context [9]. However, there is a lack of clarity regarding what makes a mental health service culturally safe. This proposed systematic review aims to identify the key characteristics of culturally safe mental health care.

The review will focus specifically on cultural safety for Aboriginal and Torres Strait Islander peoples, the First Nations people of Australia. The decision to focus on Aboriginal and Torres Strait Islander peoples was made for several reasons. First, it is not clear whether the characteristics that contribute to cultural safety for one Indigenous population would transfer to all other Indigenous populations. Therefore, this review provides a crucial and localized foundation on which future research can build upon. Second, Australia is among the leading countries driving a culturally safe healthcare agenda [10–12], therefore providing a good candidate to be a focus of this review. Further, a local focus enables the scope of this review to be feasible with the addition of grey literature to thoroughly examine, where a substantial proportion of Indigenous health research is published [13]. The review process is governed by Aboriginal researchers, and it will privilege Aboriginal and Torres Strait Islander literature and cultural knowledge, including forms of published stories, interviews, videos as this essential form of knowledge has been historically excluded.

### Cultural safety

The term *cultural* s*afety*, in the context of healthcare service provision, was first coined following observations of inequity in mainstream healthcare services in Aotearoa, New Zealand [14, 15]. More recently, this term has been applied to describe disparities in healthcare for Aboriginal and Torres Strait Islander peoples in Australia, where unsafe practices are those which diminish, demean, and/or disempower the cultural identity and wellbeing of an individual [16]. Here, we provide a description of how cultural safety is determined, which was adopted following public consultation within Australia, noting that the concept of cultural safety can be applied to any Indigenous group;

*“Cultural safety is determined by Aboriginal and Torres Strait Islander individuals*, *families and communities*. *Culturally safe practise is the ongoing critical reflection of health practitioner knowledge*, *skills*, *attitudes*, *practising behaviours and power differentials in delivering safe*, *accessible and responsive healthcare free of racism”*

[17].

Terms such as cultural safety, awareness, and competency are often used interchangeably and inconsistently [18]. However, there are some important differences between them [18–21] and this description highlights some key features which differentiate cultural safety from other similar concepts. Broadly, cultural safety is viewed as a step beyond cultural awareness and competency, because it requires more than an understanding of other cultures, and more than a set of competent-related skills. Instead, a defining feature of cultural safety, is its emphasis on recognising and responding to the critical role of power. Cultural safety for service users requires healthcare professionals and organisations to reflect on and critique taken for granted power structures, and a willingness to challenge one’s own culture, history, and privilege [18]. Importantly, cultural safety can only ever be determined by the user of a service [22]. In this context, cultural safety as a concept incorporates the idea of a changed power structure, where the power is transferred to the service user [23]. In short, cultural safety is about ensuring as far as possible, the safe journey through services and systems to achieve equitable health outcomes regardless of one’s cultural background.

### Cultural safety in mental health services

To date, reviews of cultural safety have been mostly considered in the general context of health care [18, 24], as opposed to mental health care specifically. For example, there have been reviews about culturally safe approaches in palliative care [25], diabetes [26], and health care [27] for Indigenous populations around the globe.

Cultural safety is especially important within the context of mental health services, where there are several additional complexities. For example, experiences of mental health and wellbeing are shaped by culture. Culture shapes understandings of the nature and causes of distress, the decision to seek help for distress, the kind of support that people seek, the degree of discrimination they may face, and the way diagnostic categories are constructed [28–33]. Consequently, failing to provide care that considers another’s culture can cause serious harm. For example, in some Indigenous cultures it is not unusual to experience the voices of ancestors, whereas within the Western psychiatric diagnostic system hearing voices is classified as a symptom of psychosis. Understanding whether this is a cultural phenomenon or a serious mental health disorder with a cultural dimension is critical to providing appropriate care [34]. In this context, a service which privileges Western knowledges and excludes Indigenous knowledges could potentially fail to provide culturally, and clinically safe mental health care practices, with detrimental consequences.

In Australia, the importance of cultural safety is reflected in guidelines for the Royal Australian and New Zealand College of Psychiatrists [35], accreditation standards of the Australian Medical Council [36], and national mental health policy frameworks [9], and health targets [12], specifically for Aboriginal and Torres Strait Islander peoples. However, despite growing national support for cultural safety, there is currently a lack of clarity and evidence regarding how cultural safety in mental health care can be achieved. Many mainstream mental health services fail to adequately support cultural safety [6]. Many mental health practitioners have a limited understanding of cultural safety, and do not recognise the relationship between historical events, intergenerational trauma, racism, and contemporary health inequities [6]. Thus, although the importance of cultural safety is well articulated, there is ambiguity surrounding the key characteristics which would promote or hinder cultural safety in practice.

### The systematic review

This systematic review aims to identify the key characteristics of culturally safe mental health care at the practitioner/service/system level, privileging the perspectives of Aboriginal and Torres Strait Islander peoples. When working with Aboriginal and Torres Strait Islander service users, a recent framework highlighted three principles or Aboriginal ways of Being, Knowing, and Doing [37, 38]. It is also important that cultural safety is embedded at the individual practitioner level, service level, and systems level. In this context, service providers must consider what they need to know, be, and do at individual, service and systems levels to provide culturally safe care. In our review, we will therefore consider the evidence for cultural safety at each of these levels, whenever possible.

Consistent with Indigenous research methodologies [39], a meta-synthesis approach is favourable as it enables the synthesis of qualitative studies on a topic in order to identify key themes and concepts [40]. Our review will differ to previous similar reviews [41] in that it will specifically focus on cultural safety as opposed to competency, as well as privilege the voices of Aboriginal and Torres Strait Islander peoples.

In line with best practice guidelines [42] this systematic review protocol is registered with the International Prospective Register of Systematic Reviews (PROSPERO: CRD42021258724). We undertook a preliminary search of databases (PROSPERO, PubMed, SCOPUS), and did not identify any existing or ongoing systematic reviews on the topic.

## Methods

The methodology of this qualitative systematic review (herein, meta-synthesis), was informed by the Joanna Briggs Institute Manual for Evidence Synthesis [43]. The structure of this protocol was based on the Qualitative Systematic Review protocol, using a critical and interpretive approach to synthesis and meta-aggregation [43].

### Inclusion criteria

#### Types of participants

This review will focus on Aboriginal and/or Torres Strait Islander peoples who have accessed mental health services, as well as carers, family members, and community members of the service users. There will be no restrictions on age or gender.

We will include literature published in the past 30 years (1992–2022). We describe Level 1 evidence as studies which either demonstrate improvements to cultural safety, or articles where factors which would improve cultural safety are described by Aboriginal and/or Torres Strait Islander service users (as well as carers, and/or community members of service users) of mental health services.

We consider Level 2 evidence as academic and grey literature which describe the characteristics of cultural safety (e.g., literature published by relevant Aboriginal and/or Torres Strait Islander-led organisations and services, and government policy documents). This also includes commentary, editorials, articles, and opinion pieces describing health professionals’ views of culturally safe mental health services.

Sitting alongside these two levels of evidence, we will include cultural knowledge to incorporate broader knowledges and privilege Aboriginal and Torres Strait Islander wisdom (see Table 1).

**Table 1:** Sources of evidence for the systematic review.

Level 1 Evidence	Level 2 Evidence	Cultural Knowledge
Peer-reviewed studies (qualitative) which evaluate how culturally safe a mental health service delivery was, or those which describe the key characteristics of what would make, or what made the experience culturally safe. Importantly, whether or not the service was culturally safe must come from the perspective of the service user. *Evidence may include evaluation studies with qualitative data*.	Published texts (including peer-reviewed and grey literature) which describe the characteristics of cultural safety in mental health interventions i.e., where components of a culturally safe mental health service can be extracted from a text. *Evidence may include Reports*, *Training guides*, *Any other published texts relevant to culturally safe mental health care for Indigenous peoples*. *Exclude: Literature reviews, book chapters, existing frameworks as in* [44], *to focus on ‘raw data’.*	Sources of cultural knowledge in various forms (e.g., written texts, audio recordings, video recordings). Because this literature comes from a different paradigm, we will present this side by side with other forms of evidence, with the intent of equal positioning and not comparison. *Evidence may include knowledge published from Cultural Healers*, *Elders*, *mental health service users*.

We will exclude the following: literature reviews, book chapters, and existing frameworks as in [44], to focus on ‘raw data’.

We will only conduct forward searches for papers which are included in the final review.

#### Phenomena of interest

The characteristics of a mental health intervention or service delivery which make it culturally safe.

Because the level of cultural safety of any mental health service is determined by the service user (as opposed to the service provider); see, for example, the definition of cultural safety according to the Australian Health Practitioner Registration Agency (AHPRA) [45]. Services or interventions will be considered culturally safe if they have been described as such by service users (or by carers/community members of the service user). In the literature to be reviewed, the term ‘cultural safety’ may not necessarily be used, however we will consider a service or intervention culturally safe if it is described in a way that is aligned with the definition of cultural safety used in this protocol [17].

#### Context

We will review any published intervention or service delivery which identifies, evaluates, or describes cultural safety (or similar, via a qualitative methodology; see search strategy) and designed to support the mental health or wellbeing of service users. For example, any inpatient or outpatient service delivery, mental health service, or psychiatric, psychological, psychosocial, or Social and Emotional Wellbeing intervention.

This review will consider studies that focus on qualitative data which describe the experiences or describe the effects of the experiences of cultural safety when engaging with mental health services [43]. In Table 1, the terms Level 1 and 2 types of evidence distinguish between peer-reviewed studies with an evaluation focus and other published texts with a descriptive focus.

### Search strategy

The following search terms will be used and adapted appropriately for each information source:

(Australia* and (Indigenous or Aboriginal or “Torres Strait Islander” or “First Nations” and “Cultur* Safe*”or “Cultur* Security” or “Cultur* Appropriat*” or “Cultur* Respons*” or “Cultural Framework”) and (“mental health” or Psychiatr* or Psycholog* or Psychosoc* or “social and emotional wellbeing” or suicid*) and (treatment or intervention or care or therap*or service or program or course or evaluation or trial)).

These search terms were developed and refined after preliminary searches across various databases, and a review of the key terms used in relevant literature. The key search terms identified from this method also align with the search strategy of a recent systematic review into cultural competency in the general context of healthcare (Clifford et al., 2015), confirming these terms are appropriate for our review. To be conservative, we include several search terms relating to cultural safety (e.g. cultural appropriateness, cultural responsiveness) given the inconsistent use of these terms. We will limit data to the past 30 years (1992–2022) and include sources which are in English or translated to English.

### Information sources

- PubMed, Cinahl Plus, SCOPUS, Web of Science, PsychInfo, EMBASE, Medline, Proquest, Informit, APAIS-ATSIS, and ATSIHEALTH.
- Gray Literature will be accessed from:
  - Gray literature databases (e.g., https://guides.library.cornell.edu/evidence-synthesis/gray-literature), Department of Health websites, government websites, Google, Google Scholar, Open Science Framework, Health Info Net, Grey.net.
- Search for unpublished studies will include the National Aboriginal Torres Strait Islander Health Worker Association (NATSIHWA) website (https://www.naatsihwp.org.au)
- Hand searches of the reference lists of selected articles.
- Books, videos, and other forms of publicly accessible knowledge published by Cultural Healers. These will be accessed by contacting Indigenous academics, organisations and publishing houses.

### Study selection and records

Following the search, all identified citations will be loaded into Endnote Version X9 as the mechanism for data management.

### Selection process

Selection will entail three stages:

1. Screen titles for relevance
  Two researchers will screen titles based on their relevance to the research question. For example, we will examine titles containing terms relating to mental health or social and emotional wellbeing (including terms relating to any psychological distress, stress, depression, anxiety, substance abuse, post-traumatic stress disorder, or suicide, and terms used in local languages to refer to mental health and spirit e.g., lyarn, mirin), and cultural safety (or similar, see search terms for details).
2. Screen abstracts for relevance
  Abstracts will then be assessed based on their relevance to the research question by the same two researchers, (i.e., does the article discuss the characteristics of culturally safe mental health services).
3. Screen full texts for relevance
  At this stage of the selection process, three researchers will review full texts and decide on which texts to include in the review. Number and reasons for exclusion of full text studies that do not meet the inclusion criteria will be recorded and reported in the systematic review, following the PRISMA guidelines, any discrepancies will be discussed and/or moderated by the third researcher, as described above. If discrepancies remain, the team will seek guidance from a fourth researcher, who will be a senior Aboriginal member of the team [43].

Potentially relevant studies will be retrieved in full and their citation details imported into the QSR International’s NVivo 12 qualitative data analysis software (released in March 2020).

### Data extraction

Qualitative data will be extracted by two researchers from papers included in the review using the standardized data extraction tool from JBI SUMARI by two independent reviewers. The data extracted will include specific details about the populations, context, culture, geographical location, study methods and the phenomena of interest relevant to the review question and specific objectives. Findings, and their illustrations, will be extracted and assigned a level of credibility [43]. The accuracy of data extraction will be verified by the two researchers extracting the information.

### Quality and risk of bias assessment for eligible studies

Quality and risk of bias will first be assessed by two independent reviewers according to the Aboriginal and Torres Strait Islander Quality Appraisal Tool [46]. This tool was developed by Aboriginal and Torres Strait Islander peoples, for the purposes of systematic and scoping reviews [46]. The tool consists of 14 questions, and assesses the quality of health research from an Aboriginal and Torres Strait Islander perspective [46]. This tool will be used to ensure that studies are culturally informed, i.e., researchers followed the appropriate community consultation and co-design processes, and therefore the research is of relevance to Indigenous peoples. We will report on the quality of the studies included and consider the exclusion of studies based on this criterion (e.g., if a study fails this quality check).

Authors of papers will be contacted to request missing or additional data for clarification, where required. The results of critical appraisal will be reported in narrative form and in a table [43].

### Data synthesis and integration

Data in this review will first be synthesised using a thematic analysis approach. We will then group themes according to a Framework Synthesis method, whenever possible, following the IAHA framework for Culturally Responsive practice, i.e., doing, being & knowing (as well as any other factors which are identified during the course of the review). We chose this framework as it was developed by the Indigenous Allied Health Association, and was developed to assess mental health outcomes from an Indigenous perspective.

### Ethical considerations

This research is designed and led by Aboriginal researchers and clinicians. All members of the research team (including non-Indigenous team members) will follow the guidance of cultural protocols in support of Aboriginal data sovereignty, research governance and community safety [47]. Cultural protocols referred to here include ethical guidelines for conducting research with Aboriginal and Torres Strait Islander peoples as set out by the National Health and Medical Research Council (NHMRC) of Australia [47], as well as protocols determined by the Aboriginal leadership team. Interpretation of findings for the review will be supervised by a team of Aboriginal researchers to ensure that the voices and worldviews of Aboriginal and Torres Strait Islander peoples are privleged.

## Supporting information

S1 Checklist PRISMA-P 2015 checklist. (PDF)
